# Scaling up hepatitis C testing and linkage-to-care among people who use drugs: lessons learned from a pilot project implemented at a supervised consumption site

**DOI:** 10.1186/s12913-025-12374-9

**Published:** 2025-02-13

**Authors:** Alannah Hannigan, Nandini Krishnan, Kirti Singh, Shannon Bytelaar, Deb Schmitz, Sofia Bartlett, David Hall, Rolando Barrios, Julio S. G. Montaner, Marianne Harris, Mark W. Hull, Kate A. Salters

**Affiliations:** 1 British Columbia Centre for Excellence in HIV/AIDS, Vancouver, Canada; 2 University of British Columbia, Vancouver, Canada; 3 BC Hepatitis Network, Vancouver, Canada; 4 British Columbia Centre for Disease Control, Vancouver, Canada

**Keywords:** Hepatitis C, Point-of-care, Linkage-to-care, Community, Dried-blood-spot testing

## Abstract

**Background:**

Despite rolling out publicly-funded hepatitis C virus (HCV) treatment across the province of British Columbia (BC), Canada, 35% of people returning positive HCV RNA results in 2020 did not initiate treatment. The HCV epidemic in Canada continues to disproportionately impact people who use drugs and yet, this population has the lowest proportional uptake of HCV treatment. Evidence suggests linkages to healthcare after diagnosis is one of the key factors that impacts uptake of HCV treatment among this priority population. The *Hep C Connect* pilot project was implemented to characterize HCV testing outcomes and linkage-to-care rates within a low-barrier supervised consumption site (SCS) in Vancouver, BC.

**Methods:**

All clients (aged ≥ 19 years) attending the Hope to Health SCS in Vancouver, Canada were invited to participate in the pilot study between November 2021 and December 2022. Interviewer-led surveys were conducted and participants were offered same-day HCV point-of-care (POC) antibody (Ab) testing. Participants received a cash honorarium for sharing their time and experiences. Descriptive statistics are shared in order to describe the reach and impact of this pilot project.

**Results:**

The study enrolled 186 participants including 123(66.1%) men and 59(31.7%) women, with a median age of 42 (Q1,Q3- 34,49). Forty-seven (25.3%) participants stated that they use an SCS regularly and 123(66.1%) stated that they get new rigs every day. Notably, 64(34.4%) participants reported not having a primary care provider yet more than three-quarters of the participants (144, 77.4%) reported having been ever tested for HCV. All 186 participants agreed to HCV POC Ab testing with 59.7% returning a positive HCV POC Ab result. Despite good HCV POC Ab uptake and high rates of HCV knowledge, 49(44.1%) of the HCV Ab positive participants chose not to engage in confirmatory ribonucleic acid (RNA) testing.

**Conclusions:**

The *Hep C Connect* pilot explored the gaps evident in the HCV cascade-of-care as it pertains to people who use drugs. Findings suggest that, despite high levels of HCV knowledge, the employment of blood draw RNA testing deterred people from engaging in confirmatory testing. Improving the HCV cascade-of-care will require alternative strategies that are more acceptable to this population.

**Supplementary Information:**

The online version contains supplementary material available at 10.1186/s12913-025-12374-9.

## Background

Hepatitis C virus (HCV) is a chronic viral infection that impacts approximately 58 million people globally [1]. There have been radical advancements in the treatment of HCV, including the advent of new oral direct-acting antiviral therapies that are easily tolerated and produce a cure rate of more than 95% after only 8 to 12 weeks of therapy [1]. Despite innovative policies to provide universal, publicly-funded HCV treatment across British Columbia (BC), Canada in 2018 [2, 3], the BC Centre for Disease Control reported that more than 35% of people with positive HCV ribonucleic Acid (RNA) results in 2020 did not initiate treatment [4, 5]. Research suggests that there remains a lower uptake of HCV treatment among priority population groups, including people who inject drugs, people experiencing housing insecurity, and people living with human immunodeficiency virus (HIV) [5].

The Canadian Network on Hepatitis C blueprint for HCV elimination reveals that of the 171,900 new HCV infections in Canada in 2019, 85% occurred amongst people who inject drugs, with approximately 66% of this cohort currently or previously having had HCV infection [6]. In Canada, the HCV epidemic continues to disproportionately impact people who use drugs (PWUD) (a diverse group of individuals who use unregulated substances) and yet, it is precisely this population that has the lowest proportional uptake of HCV treatment [6, 7]. Individual, systemic and social barriers, comorbid medical conditions, misconceptions about HCV as well as healthcare-related stigma are all seen to contribute to low treatment uptake among this population [8, 9].

Literature suggests PWUD engage with the healthcare system by means of emergency services and hospitalizations, indicating a lack of linkage to and engagement with primary healthcare [9, 10]. Critically, the lack of *supportive* linkages to primary healthcare offers an explanation for the poor uptake of HCV treatment among PWUD [11–14]. While some clients may be referred to specialists to manage HCV care and treatment, social and structural barriers such as opening hours, geographical location, proximity and cost-to-attend may hinder PWUD ability to make it to these specialist appointments [11, 15]. Healthcare-related trauma is often overlooked as another explanation for poor uptake of HCV treatment. This trauma can be seen to impact the development of future relationships between PWUD and healthcare, once again effecting treatment rates and more general healthcare engagement [16].

A reactive healthcare system that continues to offer only mainstream treatment and care approaches for clients with complex health and psycho-social needs could be seen to perpetuate health inequities, and to work in direct contradiction to the 2016 World Health Organization roadmap to eliminate viral hepatitis by the year 2030 [17].

Supervised consumption sites (SCS) and other harm reduction services are instrumental in supporting and improving care delivery for PWUD. These sites have been operating for decades in Canada, and sanctioned by the federal body Health Canada, as a way to provide safe and clean spaces for people to bring their own drugs to use, in the presence of trained staff [18]. These services save lives by preventing accidental overdoses, while also reducing the spread of infectious diseases and have a positive impact on the health system [19–22]. Beyond providing a supervised space for unregulated drug consumption, SCSs also provide clients with harm reduction supplies, overdose prevention strategies, informational support regarding safer usage, and often link clients to other sources of support [23]. SCSs and harm reduction sites also often employ people with lived and living experiences (Peers) to help build on interpersonal relationships and guide client engagement. Research shows that consistent SCS engagement has been positively associated with safer usage and healthier behaviours even outside the SCS setting [19, 24, 25]. Much of this is due to the low-barrier nature of SCS access and engagement. Many SCS and harm reduction sites have used this lower-barrier setting and expanded their services to include additional healthcare services for their clients. Encouragingly, this practice has proven to be especially helpful in improving linkage-to-care amongst PWUD [26]. However, despite these developments, harm reduction is still often seen as something that is happening alongside, and that is distinctly different from, healthcare [27, 28]. This separation of healthcare and harm reduction may not be serving the needs of PWUD.

Working towards higher HCV testing rates and increased linkage-to-care among PWUD, the *Hep C Connect* pilot project was launched in a low-barrier SCS located in the downtown eastside (DTES) neighbourhood of Vancouver, BC. The study objective was to evaluate the impact of a nurse-led intervention facilitating linkage-to-care for participants who were unattached to primary care, yet accessing harm reduction services. In this paper, we describe the cohort of clients reached by the *Hep C Connect* pilot project, outcomes of HCV testing and linkage-to-care, as well as lessons learned and implications for the future.

## Methods

### Study population

The *Hep C Connect* study was carried out at the British Columbia Centre for Excellence in HIV/AIDS Hope to Health (H2H) complex in the DTES. One of Vancouver’s oldest neighbourhoods, the DTES is a vibrant and diverse community with a dynamic history and a strong sense of resilience amongst its residents. Faced with challenges of homelessness, mental health and substance use it is often characterized by its high prevalence of social marginalization [29]. Launched in 2019, the H2H complex is an interdisciplinary primary care service that features a co-located primary care clinic, clinical and behavioural research team, a clinical research laboratory and an SCS [30].

Funding for the pilot was provided by Gilead Sciences through a community initiative grant that provided funding for study administration and OraQuick HCV rapid point-of-care (POC) antibody (Ab) testing kits in-kind (2021–2022). This project was approved by the UBC and Providence Health Care Research Institute (REB #H21-01534).

### Intervention

Clients of the H2H SCS were eligible to join the study if they were ≥ 19 years of age, willing to provide informed consent, and able to verbally communicate through English. All eligible clients of the H2H SCS were invited to participate in the *Hep C Connect* pilot project from November 2021-December 2022. The sample is comprised of a diverse group of PWUD who had accessed the H2H harm reduction service and informed consent to participate in the study was obtained from all participants. For all eligible participants enrolled and consented into the study, the research team offered HCV POC Ab testing and invited them to participate in an in-person, interviewer-administered survey lasting approximately 30-min. Participants received a cash honorarium of thirty-dollars at the end of each study visit. Participants were informed that their ability to participate and receipt of the honorarium was not contingent on engagement in the HCV POC Ab testing and those that chose not to do any testing would still receive an honorarium for their time and contribution to the research study.

Clinical follow-up for all study participants was initiated by the clinical research nurse, including offering on-site confirmatory blood tests (HCV RNA) via blood draw, and supportive linkage to a low-barrier primary healthcare clinic (co-located at H2H) for those unattached to care. For those already attached to primary care elsewhere (i.e., another community healthcare centre (CHC)), their results were faxed and/or mailed to their primary care provider so as not to disrupt their continuity of care. The HCV POC Ab testing, study survey, and confirmatory RNA blood draw (if required) were conducted on-site at the H2H SCS on the same day. The HCV RNA sample processing time was approximately one-week. Participants who returned a positive HCV RNA result and indicated no existing attachment to primary care were subsequently offered the referral to the H2H primary care clinic. If a participant was already attached to a different primary care clinic, as stated the research nurse communicated the results with the participant’s primary healthcare provider in order for the participant and their provider to be able to make an informed decision about their follow-up care and treatment.

### Data collection

Baseline data were collected between November 2021 and December 2022 using standardized interviewer-led surveys. Follow-up data were collected in 2023. This analysis utilizes baseline data only. To fulfill our study objectives, our survey tool (developed specifically for this research study) collected self-reported demographic information as well as information on recent healthcare utilization, harm reduction engagement, and substance use, as well as HCV testing and treatment history and HCV knowledge. Interviews were conducted in-person by one of the trained research team members at the H2H SCS. The study engagement was comprised of a baseline survey of approximately 30-min in length (including wait time for the HCV POC Ab test) and two subsequent follow-up surveys at 3 and 12-months that were 10–15 min in length. If participants were HCV RNA positive, they were asked questions about their experience with HCV care and treatment in their follow-up surveys.

### Variables

Participants were encouraged to complete each survey in its entirety, however, as per the study consent agreement, there was the option to skip questions and to end the survey early should the participant choose to. Within the survey, we used psychometrically validated scales to assess participants relationships with their healthcare providers. Specifically, we used the Healthcare Trust scale as a means to understanding patient and healthcare provider relationships. We also employed an 8-item validated HCV knowledge scale in order to better understand participants understanding of HCV.

For the purposes of this analysis, we only used the baseline survey data. Variables such as age (median), gender (cis and trans men, cis and trans women, non-binary or Two Spirit individuals), sexual identity (straight and lesbian, gay, bi-sexual, queer, asexual (LGBQA +)), ethnicity (Indigenous, white and other racialized community), country of origin (Canada and outside of Canada), history of incarceration (ever and never), housing and income were collected and measured through the survey tool.

### Statistical analysis

Descriptive statistics (i.e., mean, median, standard deviation, and proportions) were used to characterize the study cohort. In this analysis, we characterize our cohort’s HCV testing history, harm reduction behaviours and healthcare engagement. This is assessed overall, and stratified by participants attached to the H2H primary care clinic, participants who are linked to other CHCs, or participants who are unattached to primary healthcare entirely (i.e., individuals disengaged and unattached to primary care in the past 12-months). Finally, we characterize a cross-sectional snapshot of the HCV cascade-of-care in this cohort.

## Results

The *Hep C Connect* pilot enrolled 186 participants (Table 1). The median age of participants was 42 [31], 59(31.7%) identified as women, and 81(43.6%) identified as Indigenous.

**Table 1:** Characteristics of the study cohort (*N* = 186)

**Variables**		**Median (IQR) or n (%)**
Age	Median (IQR)	42(34,49)
Gender	Women	59(31.7%)
	Men	123(66.1%)
	Two Spirit and/or Non-binary	< 5
Sexual Identity	Straight	158(84.9%)
	LGBQA +	28(15.1%)
Ethnicity	Indigenous	81(43.6%)
	White	94(50.5%)
	Other (Black, East Asian, South Asian)	11(5.9%)
Country of Origin (born)	Canada	176(94.6%)
	Other (Outside of Canada)	10(5.4%)
Ever been incarcerated	Yes	155(83.3%)
	No	31(16.7%)
Housing in the past 3-months (not mutually exclusive)	Homeless	72(38.7%)
	Hotel/SRO	43(23.1%)
	Other (Supportive housing, Subsidized housing, Market rental and other)	74(39.8%)
Income in the past 3-months (not mutually exclusive)	Full/Part-time job	20(10.8%)
	Stipend	17(9.1%)
	Disability assistance	93(50%)
	Income assistance	76(40.9%)
	Recycling/binning	42(22.6%)
Primary care status (at baseline)	Attached to H2H	47(25.3%)
	Attached to care elsewhere	75(40.3%)
	Unattached to care	64(34.4%)

**Fig. 1:**
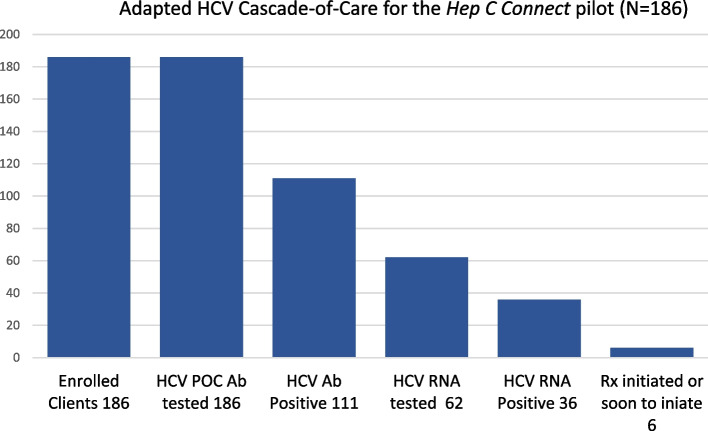
Adapted HCV cascade-of-care for the *Hep C Connect* pilot (*N* = 186)

Results show that 47(25.3%) participants reported already being connected to care at the H2H clinic, 75(40.3%) reported attachment to other CHCs while the remaining 64(34.4%) reported not having a primary healthcare provider. Progress across the HCV cascade-of-care (defined as including the following stages: Enrolled, HCV POC Ab tested, HCV Ab positive, HCV RNA tested, HCV RNA positive and HCV treatment initiated) was assessed, in turn producing an Adapted HCV Cascade-of-Care for the *Hep C Connect* pilot (Fig. 1).

A history of incarceration (ever) was reported by 155(83.3%) of participants while more than half of the participants (*N* = 114, 61.3%) reported *ever* experiencing homelessness. When discussing harm reduction engagement and behaviors, 47(25.3%) stated that they use SCSs every day with 123(66.1%) stating that they get new rigs every day at least once a day. Importantly, 69(37.1%) participants reported using drugs alone all of the time and 35(18.8%) reported using drugs alone most of the time (several times per week). More than three quarters of the participants (*N* = 144, 77.4%) reported having been tested for HCV *ever*, while 93(50%) reported having been tested in the last five years.

When asked about engagement in primary healthcare, 64(34.4%) participants reported not having a primary care provider, 47(25.3%) reported being connected to care at the H2H clinic while the remaining 75(40.3%) reported attachment to other primary healthcare providers. Significantly, 26.9% of participants asked (*n* = 135) had not been to visit a primary healthcare provider in more than a year. When speaking about avoidance of primary healthcare engagement, 25(18.5%, *n* = 135) stated anticipation of inadequate care as the reason for avoiding going to the doctor in the past 12-months.

**Fig. 2:**
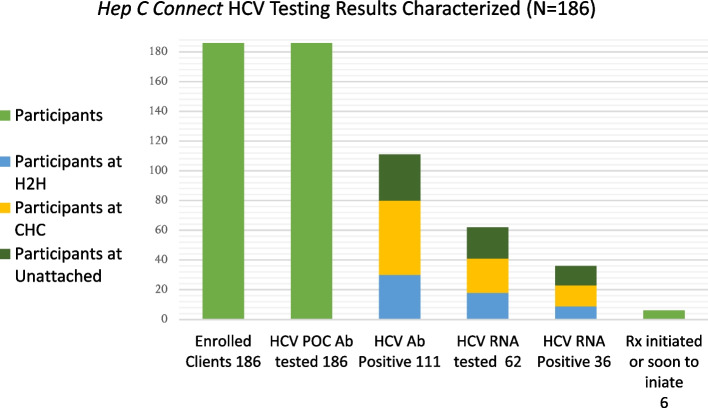
*Hep C Connect* HCV testing results characterized

HCV POC Ab testing in the pilot study found a substantial proportion of participants were HCV Ab positive. All 186 participants engaged in the HCV POC Ab testing (186/186); 111/186(59.7%) participants had a reactive HCV Ab test, of whom 62/111(55.9%) chose to engage in HCV RNA blood draw testing, and 36/111(32.4%) returned a positive HCV RNA result. The adapted HCV cascade-of-care can be seen in Fig. 1 while the characteristics of participants with positive HCV Ab results can be seen in Table 2 and Fig. 2***.***

**Table 2:** Characteristics of participants who had positive HCV Ab results (*N* = 111)

**Variables**		**HCV Ab + **Attached to care at H2H (%)	**HCV Ab + ** Attached to care elsewhere (%)	**HCV Ab + ** Unattached to care (%)	**Total**
Gender	Women	10(9.0%)	15(13.5%)	7(6.3%)	32
	Men	20(18.0%)	33(29.7%)	23(20.7%)	76
	Two-Spirit and/or Non-binary	< 5	< 5	< 5	< 5
Sexual Identity	Straight	30(27.0%)	40(36.1%)	26(23.4%)	96
	LGBQA +	< 5	10(9.0%)	< 5	15
Ethnicity	Indigenous	12(10.8%)	29(26.1%)	10(9.0%)	51
	White	17(15.3%)	19(17.1%)	18(16.2%)	54
	Other (Black, East Asian, South Asian)	< 5	< 5	< 5	6
Ever been incarcerated	Yes	27(24.3%)	45(40.6%)	26(23.4%)	98
	No	< 5	< 5	< 5	13
Ever experienced home-lessness	Yes	13(11.7%)	29(26.1%)	21(18.9%)	63
	No	17(15.3%)	21(18.9%)	10(9.0%)	48
Use substances alone	Never **or** Rarely	9(8.1%)	20(18.0%)	7(6.3%)	36
	Occasionally **or** Some of the time	< 5	8(7.2%)	< 5	14
	Most **or** All of the time	18(16.2%)	22(19.8%)	21(18.9%)	61
Use SCSs/ overdose prevention sites(OPSs)	Never **or** Rarely	11(9.9%)	10(9.0%)	8(7.2%)	29
	Occasionally **or** Some of the time	< 5	< 5	6(5.4%)	16
	Most **or** All of the time	14(12.6%)	35(31.5%)	17(15.3%)	66

## Discussion

The *Hep C Connect* pilot effectively engaged a cohort of PWUD at high risk of HCV. Of note, all of the study participants (*N* = 186) chose to engage in HCV POC Ab testing, and 111(59.7%) returned a reactive HCV Ab test. This highlights the importance and impact of low barrier POC testing, which allows for earlier intervention, more convenience for patients, as well as improved turn-around time of results, all of which have been seen to contribute to improved treatment follow-up [32]. It is also worth noting that participants of this study were already clients of the H2H SCS and therefore had experience with and were familiar with the staff and setting within which this testing was being offered. This, along with the low barrier HCV POC Ab testing could be seen to positively impact engagement among this cohort. POC is also attractive as it is less invasive, requiring small lancets, and a smaller specimen [33].

A 2023 systematic review examined how POC viral load testing could enhance HCV detection and other key outcomes along the care cascade [34]. Compared to laboratory testing, onsite testing saw an overall increase in treatment uptake as well as reduced turnaround times between HCV testing and treatment initiation [34]. The review also noted an increase in HCV viral load testing and treatment uptake with the use of POC viral load testing noting the greatest increase among the cohort of people who inject drugs [34]. Additionally, literature suggests HCV knowledge, specifically among people who inject drugs has been associated with greater HCV treatment willingness [31]. HCV knowledge was high amongst participants in the *Hep C Connect* study (median score of 7/8 correct responses). Of the participants who were asked (*N* = 135) 86.3% knew that HCV primarily impacts the liver; 80.8% correctly answered that HCV can only be diagnosed by means of a blood test; 94.5% knew that untreated HCV can result in liver failure, and 91.8% knew there is a curative treatment for HCV. Engagement with healthcare within this cohort was also positively associated with increased HCV knowledge.

Research indicates that despite high rates of testing, linkage-to-care (i.e., clinical management withing 6 months of diagnosis) remains particularly low among people who use substances [35]. Despite the *Hep C Connect* study observing high levels of HCV knowledge among the study cohort and too, high levels of HCV POC Ab testing, many participants chose to opt out of RNA confirmatory testing. As a result of this, there is no confirmation that these participants continued on to primary care treatment and/or became linked to care.

Forty-nine (44.1%) of the Ab positive participants chose not to engage in blood draw confirmatory testing. Fear of diagnosis, stigma, antipathy regarding mainstream healthcare services and other competing priorities (i.e.: substance use, other healthcare issues, time and accessibility) have been shown to deter PWUD from engaging in HCV testing [36]. Many participants anecdotally noted an inability to manage an HCV diagnosis due to social factors such as unstable housing. Participants also stated how their physical symptoms, or lack thereof, for HCV did not warrant the discomfort of confirmatory testing. Literature confirms that this substantial loss to follow-up persists across the HCV care continuum and is especially evident among underserved populations [37–39]. Alternative specimen types, such as dried-blood-spots (DBS) or capillary tubes that can be collected using finger-poke rather than venepuncture, have been shown to be an effective strategy to increase uptake of confirmatory testing and HCV treatment among PWUD [40]. Expansion of access to these specimen types in primary care and community settings such as SCSs and OPSs is warranted, given the observed high HCV Ab positivity and low uptake of confirmatory testing observed here.

### Limitations

We acknowledge that the pilot study has limitations. First, self-report respondent bias adherent to self-report studies, such as social desirability bias is possible. In order to minimize this type of bias, our data collection was carried out using trained and skillful interviewers, as well as using psychometrically validated scales. Secondly, the pilot study consisted of 186 participants. Although somewhat small in scale, this study is very timely considering the continued increase in HCV among the underserved population of the DTES and limited research in this area to guide policy. Thirdly, we recognise the impact of employing blood draw RNA confirmatory testing. As the pilot demonstrated, participants were willing to engage in HCV POC Ab testing but where less inclined (*N* = 49, 44.1%) to engage in the blood draw RNA confirmatory testing. This in turn impacted one of the pilots overarching objectives to connect participants to care with the goal of improving the HCV cascade-of-care.

### Future considerations

Beginning January 2024, we have expanded our data collection with 300 new participants and have initiated a full-scale intervention based on the *Hep C Connect* pilot. Our evaluation will assess the efficacy of an integrated service delivery of harm reduction and HCV care in addressing treatment disparities and researching patients who face barriers to care. In order to better facilitate our participants needs, and learn from the success of the POC engagement in the pilot, we will move to employ DBS testing as a means to improve HCV RNA confirmatory testing uptake and reduce barriers to care. This will also allow the study to broaden its objectives and facilitate DBS testing to include HIV and syphilis alongside HCV.

## Conclusion

The *Hep C Connect* pilot addresses the current gaps in the HCV cascade-of-care in BC with a particular focus on HCV testing and linkage-to-care. By optimizing the uniquely co-located primary healthcare clinic and H2H SCS, our nurse-led intervention of HCV POC Ab testing centres the patient’s needs working to improve engagement and positively impact the HCV cascade-of-care. Importantly, expansion of the project will broadly allow for more education, awareness and openness about HCV and sexually transmitted blood borne infections within a community-based service for PWUD. Additionally, the project will once more prioritize individuals that have been previously lost to follow-up and work to improve overall linkage-to-care and care continuity in the DTES of Vancouver.

## Supplementary Information

Supplementary Material 1.

## Data Availability

The BC Centre for Excellence in HIV/AIDS is prohibited from making individual-level data available publicly owing to provisions in our service contracts, institutional policy, and ethical requirements. To facilitate research, we make such data available via data access requests. Some data from the Centre are not available externally owing to prohibitions in service contracts with our funders or data providers. Institutional policies stipulate that all external data requests require collaboration with a researcher from the BC Centre for Excellence in HIV/AIDS. For more information or to make a request, please contact Mark Helberg (mhelberg@bccfe.ca).

## Abbreviations

HCV: Hepatitis C virus
BC: British Columbia
RNA: Ribonucleic acid
HIV: Human immunodeficiency virus
PWUD: People who use drugs
SCS: Supervised consumption site
DTES: Downtown eastside
H2H: Hope to Health
POC: Point-of-care
Ab: Antibody
CHC: Community healthcare centre
LGBQA +: Lesbian, gay, bi-sexual, queer, asexual
OPS: Overdose prevention site
DBS: Dried-blood-spot
