# Supplementary material for: Scaling up hepatitis C testing and linkage-to-care among people who use drugs: lessons learned from a pilot project implemented at a supervised consumption site
Source: BMC Health Serv Res. 2025 Feb 13;25:243. doi: 10.1186/s12913-025-12374-9 (PMC11823232; doi:10.1186/s12913-025-12374-9)
Supplement: Supplementary file 1 — Supplementary Material 1. [file 12913_2025_12374_MOESM1_ESM.docx]

Hep C Connect

Baseline Survey

Section1: Demographics

1. **What is your date of birth?**

Day/month/year

1. **How would you describe your gender identity?**
2. Woman

- Cisgender
- Transgender
- Other

1. Man

- Cisgender
- Transgender
- Other

1. Non-binary
2. Other
3. Prefer not to answer
4. **What term best describes your sexual orientation? (Check all that apply)**
5. Straight
6. Gay
7. Lesbian
8. Bisexual
9. Queer
10. Asexual
11. Other
12. Prefer not to answer
13. **A) Do you identify as an Indigenous person (First Nations, Inuit or Metis)?**
14. Yes
15. No (If no, go to question 5)
16. Prefer not to answer

**B) If you identify as an indigenous person, are you (Check all that apply)**

1. First Nations
2. Metis
3. Inuit
4. Other (please specify)
5. Not applicable

**C) If you do identify as Indigenous, do you identify as Two Spirit?**

1. Yes
2. No
3. Prefer not to answer
4. Not applicable
5. **Which category(ies) best describes you? (Check all that apply)**
6. African, Caribbean or Black
7. White
8. South Asian
9. Chinese
10. North African/West Asian or Arab
11. Filipino
12. Korean
13. Latin American
14. South Asian
15. Japanese
16. Other/mixed race
17. Prefer not to answer
18. **Where you born in Canada?**
19. Yes
20. No

- Country of Origin
- Year moved to Canada

1. Prefer not to answer
2. **What is your current relationship status?**
3. Marries
4. Living common Law
5. Dating (Steady partner or in a relationship)
6. Separated
7. Single
8. **Have you ever been incarcerated (jail or prison)?**
9. Yes

- Yes, In the past 12 months
- Not in the past 12 months

1. No
2. Prefer not to answer
3. **Can you describe your housing in the past 3 months? (Check all that apply)**
4. Homeless/ NFA (Check all that apply)
5. Primary (sleeping outside/in a tent)
6. Secondary (couch surfing/ staying with a friend)
7. Tertiary (insecure housing e.g. shelter)
8. Hotel/SRO
9. Supportive Housing (e.g., transitional housing)
10. Subsidized housing (e.g., BC Housing)
11. Market rental
12. Other (specify)
13. **Are you currently receiving any government assistance? (If yes, check all that apply)**
14. Yes

- Persons with disability (PWD) benefit
- Income assistance (or “welfare”)
- Workers’ compensation (WCB)
- Employment insurance (“EI”)
- Persons with persistent multiple barriers (PPMB)
- Canada Pension Plan (CPP)
- Old age security (OAS)
- Other(specify)
- Not applicable

1. No
2. Prefer not to answer
3. **A) In your day-to-day life, on a scale of 1-10 (1 being not supported at all and 10 being very supported) how supported do you feel?**
4. 1 2 3 4 5 6 7 8 9 10

**B) Who is the person(s) that supports you? (Check all that apply and indicate most important)**

1. Friend
2. Family member
3. Romantic partner/spouse
4. Roommate
5. Pet
6. Healthcare worker
7. Community support worker
8. Social worker
9. Other (specify)
10. Myself
11. Don’t know
12. No one

Section 2: Harm Reduction

1. **In the past 3 months, which of the following substances have you used? (Check all that apply in each category)**

For each drug indicated yes: injection? (y/n); inhalation? (y/n); daily use? (y/n)

1. Cannabis (marijuana, pot, grass, harsh, etc.)
2. Cocaine (coke, crack, etc.)
3. Amohetamine-type stimulants (speed, meth, ecstasy, etc.)
4. Inhalants (nitrous, glue, petrol, paint thinner, etc.)
5. Sedatives or sleeping pills (diazepan, alprazolam, flunitrazepam, midazolam, etc.)
6. Hallucinogens (LSD, acid, mushrooms, trips, ketamine, etc.)
7. Opioids (heroin, morphine, methadone, buprenorphine, codeine, etc.)
8. Other (specify)
9. **A) Do you ever use alone?**
10. Yes
11. No
12. Prefer not to answer

**B) If yes, how often in the past 3 months did you use alone?**

1. Rarely (less than once per month)
2. Occasionally (couple times per month)
3. Some of the time (once per week)
4. Most of the time (several times per week)
5. All of the time (every day)
6. Don’t know
7. Prefer not to answer
8. **A) Do you ever use supervised consumption sites?**
9. Yes
10. No
11. Prefer not to answer

**B) If yes, how often in the past 3 months did you use supervised consumption sites (or OPS)?**

1. Rarely (less than once per month)
2. Occasionally (couple times per month)
3. Some of the time (once per week)
4. Most of the time (several times per week)
5. All of the time (every day)
6. Don’t know
7. Prefer not to answer
8. **Which of the following SCS/OPS sites have you used in the past 3 months? (Check all that apply)**
9. Insite
10. Molson OPS (*Hastings St. alley*)
11. Thomus Donaghy OPS
12. OPS (Columbia Street)
13. OPS Inhalation tent
14. Powell St. Getaway
15. Sister Space
16. Sister Space Inhalation Tent
17. H2H
18. Other (specify)
19. Not applicable (I do not use SCS/OPS sites)
20. **Can you describe some of the reasons why you might choose not to use at or are unable to use at an SCS/OPS? (Check all that apply)**
21. Wait time / line up too long
22. Too far from my residence / too far in general
23. I couldn’t wait to use (e.g., was dope sick)
24. SCS / OPS not open when I need
25. I was barred / suspended
26. I was uncomfortable with other SCS / OPS clients
27. I was uncomfortable with SCS/OPS staff
28. I prefer to use at home
29. I prefer to use alone/ in private
30. I do not like the SCS/OPS rules (e.g., sharing with other clients, etc.)

| 1. Not enough inhalation services |
| --- |
| 1. Too cold to use outdoor inhalation tent |
| 1. Other (specify) |
| 1. Prefer not to answer |
| 1. Not applicable |

1. **In the past 3 months, who are the people you have used with when not using alone or at a SCS/OPS? (Check all that apply and check most frequent)**
2. Friend/ acquaintance
3. Family member
4. Partner (co-habitating)
5. Partner (not co-habitating/together longer than 3 months)
6. Casual partner (dating/less than 3 months)
7. Roommate
8. Dealer
9. Stranger
10. Other (specify)
11. Not applicable; I have not used in the past 3 months
12. Not applicable; I always use alone when not using an SCS
13. Prefer not to answer
14. **How often did you get new rigs and equipment in the past 3 months?**
15. More than once a day
16. Every day
17. Every couple of days
18. Once a week
19. Every couple of weeks
20. Once a month
21. Less than once a month
22. Don’t know
23. Prefer not to answer
24. **A) In the past 3 months, have you fixed with a rig that had been used by someone else?**
25. Yes
26. No
27. Unsure
28. Not applicable
29. Prefer Not to answer

**B) If yes, who was the person?**

1. Partner
2. Friend/acquaintance
3. Stranger
4. Other (specify)
5. Unsure
6. Not applicable
7. Prefer not to answer
8. **A) In the last 3 months, have you used a rig to split your drugs with another person?**
9. Yes
10. No
11. Unsure
12. Not applicable
13. Prefer not to answer

**B) If yes, who was the other person? (Check all that apply)**

1. Partner
2. Friend/acquaintance
3. Stranger
4. Other (specify)
5. **A) Are you currently using any prescription such as OAT or Safe supply for OUD?**

OAT

1. Yes oat
2. No oat
3. Prefer not to answer

Safe Supply

1. Yes, safe supply
2. No safe supply
3. Prefer not to answer

**B) If yes, indicate for each substance: current dose/how satisfied/reasons why**

1. Methadone/Methadose
2. Suboxone
3. Hydromorphone

- Oral
- Injection

1. Kadian
2. M-Eslon
3. Fentanyl patch
4. Fentanyl tablet
5. Diacetlymorphine
6. Other (specify)
7. **A) Have you encountered challenges in accessing prescription treatment for OUD (OAT or Safe Supply)**
8. Yes
9. No
10. I do not want prescription treatment for OUD
11. Not applicable (not an opioid user)
12. Prefer not to answer

**B) If yes, please indicate why (check all that apply)**

1. Unable to find a provider/clinic
2. Waitlisted
3. Unsure of how to access
4. Denied treatment
5. Kicked out of a program
6. Barred from clinic
7. Available options do not work for me
8. Program schedule is a barrier
9. I had a negative reaction to a prescription
10. Discouraged as a result of negative experiences/stigma with healthcare providers
11. Other (specify)
12. **Have you been sexually active (meaning oral, anal or vaginal) in the past 3 months?**
13. Yes
14. No
15. Unknown
16. Prefer not to answer
17. **When you have sex, what is the gender(s) of the person(s) that you have sex with?**
18. Men
19. Women
20. Non-binary
21. Unknown
22. Prefer not to answer
23. Not applicable
24. **Have you been tested for syphilis, HIV or other STIs in the past 3 months?**
25. Yes (specify)
26. No
27. Unknown
28. Prefer not to answer
29. **Have you heard of pre-exposure prophylaxis (PreP)?**
30. Yes
31. No
32. Unsure
33. **Would you like to know more about accessing PreP?**
34. Yes
35. No
36. Unsure

Section 3: Healthcare

1. **Do you have a primary healthcare provider?**
2. Yes
3. No
4. **A) What healthcare providers have you seen in the past 3 months? (Check all that apply)**

**B) Who do you consider to be your main (most important) primary healthcare provider? (Check only one)**

1. None
2. GP
3. Nurse
4. Social worker
5. Outreach worker
6. Elder
7. Peer navigator
8. Specialist (specify)
9. Pharmacist
10. ER doctor
11. ER Nurse
12. Other (specify)
13. **How often do you meet with them (most important provider)?**
14. More than once a week
15. Once a day
16. Several times per week
17. Once a week
18. Every 2 weeks
19. Once a month
20. Less than once a month
21. Not applicable
22. **Do you always go to the same clinic for primary health care?**
23. Yes
24. No
25. Unknown
26. Other (specify)
27. Not applicable
28. **What best describes the type of primary care facility that you access?**
29. Walk-in clinic
30. Family doctors practice
31. Community health centre (i.e., Pender, DCHC)
32. Community agency
33. Methadone clinic
34. Other (specify)
35. **Do you always see the same primary care provider at your primary care facility?**
36. Yes
37. No
38. Unknown
39. Other (specify)
40. Not applicable
41. **Do you feel you are ever judged or stigmatized by your current primary care provider for using drugs?**
42. Never
43. Sometimes
44. Often
45. Always
46. Don’t know
47. Not applicable
48. **A) Is healthcare currently meeting your needs?**
49. Yes
50. No
51. Unsure
52. Prefer not to answer

**B) Do you think any of your healthcare needs are being neglected (Check all that apply)**

1. Mood and/or anxiety disorders
2. Psychosis or Schizophrenia
3. PTSD/trauma
4. Drug use or addiction
5. Infections/wounds
6. Alcohol use
7. Heart disease
8. High blood pressure
9. COPD or respiratory disease
10. Diabetes
11. Being overweight
12. Being underweight
13. Chronic pain
14. Physical disability or injury
15. HIV
16. Hepatitis C
17. Sexual Transmitted Infections
18. Other (specify)
19. A**) How long has it been since your most recent visit with a primary care provider? (*e.g., a family doctor that the participant sees on a semi regular basis for general health concerns*)**
20. Days:/Weeks:/Months:/Years:
21. Unknown – Can’t remember last visit
22. Prefer not to answer

**B) Please indicate the position of the primary care provider being referenced.**

1. Pharmacist
2. Family Doctor
3. Nurse
4. Social Worker
5. Other (specify)
6. **In the past 12 months, have you avoided going to the doctor?**
7. Yes
8. No
9. Prefer not to answer
10. **What are some of the reasons you have avoided going to the doctor in the last 12 months? (Check all that apply)**
11. No reason to see a doctor
12. Not a priority for me
13. Doctor talks down to me
14. Doctor is judgmental
15. Waiting for the issue to resolve itself
16. Long wait times
17. Too much travel time
18. Inadequate care previously
19. Anticipation of inadequate care
20. Care is not culturally appropriate
21. Other (specify)
22. Don’t know
23. Not applicable
24. **Have you experienced any of the following in relation to finding a family doctor? (Check all that apply)**
25. Not a priority for me
26. Need help locating one/unsure where to look
27. Waitlisted
28. Can’t find clinic accepting new patients
29. Can’t find a doctor I like
30. None in my preferred area
31. NA; I have a doctor
32. NA; I don’t need one
33. Other (specify)
34. **Do you feel you can openly speak to your current primary care provider about your personal goals and needs when it comes to substance use?**
35. Never
36. Sometimes
37. Often
38. Always
39. Don’t know
40. Not applicable
41. **A) Have you thought about changing your current primary health care provider?**
42. Yes-ever
43. Yes-in the past 3 months
44. No
45. Unknown
46. Prefer not to answer
47. Not applicable

**B) Why have you thought about changing your healthcare provider (Check all that apply)**

1. Unsatisfied with care
2. Received a recommendation from someone else
3. Access issues (transportation, travel time, etc…)
4. Difficulty getting appointments
5. Negative/unpleasant relationship with provider
6. Negative/unpleasant interactions with other clinic staff
7. Other (specify)
8. **Have you been hospitalized or visited an emergency room for any reason in the past 3 months? (Check all that apply)**
9. Yes (number of times)

- Pneumonia
- Heart Issues
- Injury
- Overdose
- Skin infection/wound
- COVID
- Psychiatric: depression, suicidality, etc.
- Psychiatric: psychosis
- Can’t recall
- Other (specify)

1. No
2. Unknown
3. Prefer not to answer
4. **Have you ever been tested for HCV before?**
5. Yes - finger prick
6. Yes- RNA
7. Yes-can’t recall details
8. Unsure
9. No
10. Prefer not to answer
11. **In the past 1 year, can you recall being offered any form of HCV testing (e.g. community event, outreach, study, etc.)**
12. Yes

- Clinic or CHC
- Study (e.g. – VIDUS)
- SCS or OPS
- Hospital
- Community organization (e.g. – Dr. Peter Centre)
- Community event
- Drop-in Centre
- Outreach team
- Other (specify)

1. No
2. Unknown
3. **Have you ever been told you have hepatitis C?**
4. Yes -Active
5. Yes- Cleared
6. Yes - Treated
7. No
8. I don’t know
9. Prefer not to answer
10. **Have you ever taken treatment for hepatitis C infection?**
11. Yes; what medication were you offered? (Check all that apply)

- Interferon
- DAAs
- Unsure/ can’t recall

1. No
2. I don’t know/remember
3. **A) How would you assess your risk for getting hepatitis C currently?**
4. High
5. Moderate
6. Low
7. Zero
8. I don’t know
9. Not applicable

**B) Explain:**

1. **Thinking back to the past 1 year, has your current (or most recent) health care provider ever discussed/offered any of the following? (Check all that apply)**
2. Information about what HCV is
3. Information around HCV transmission risks
4. Prevention measures (e.g., harm reduction practices) that protect against HCV
5. Discuss HCV treatment options
6. Offered an HCV test
7. Followed up with interest in HCV treatment
8. Provided end of treatment testing
9. Other general HCV related info
10. My current/most recent provider hasn’t discussed HCV with me
11. I don’t remember
12. **Have you received Hepatitis A or B vaccine (from your provider or elsewhere)?**
13. Yes, Hep A
14. Yes, Hep B
15. Yes, Hep A & B
16. No, neither
17. I don’t remember
18. Prefer not to answer

Everyday discrimination scale (EDS) Short version *(Check one box per line)*

|  | Never | Less than once a year | A few times a year | A few times a month | At least once a week | Almost every day |
| --- | --- | --- | --- | --- | --- | --- |
| 1. **You are treated with less courtesy or respect than other people** |  |  |  |  |  |  |
| 1. **You receive poorer service than other people at restaurants or stores** |  |  |  |  |  |  |
| 1. **People act as if they think you are not smart** |  |  |  |  |  |  |
| 1. **People act as if they are afraid of you** |  |  |  |  |  |  |
| 1. **You are threatened or harassed** |  |  |  |  |  |  |

1. **What do you think is the main reason for these experiences? (Check all that apply) (Asked only of those answering “A few times a year” or more frequently to at least one question.)**
2. Your Ancestry or National Origins
3. Your Gender
4. Your Race
5. Your Age
6. Your Religion
7. Your Height
8. Your Weight
9. Some other Aspect of Your Physical
10. Appearance
11. Your Sexual Orientation
12. Your Education or Income Level
13. A physical disability
14. Your shade of skin color (NSAL)
15. Your tribe (SASH)
16. Other (specify)
17. Not applicable

Healthcare relationship (HCR) trust scale *(Check one box per line)*

|  | None of the time | Some or a little of the time | Occasionally or a moderate amount of the time | Most of the time | All of the time |
| --- | --- | --- | --- | --- | --- |
| 1. **How often does your health care provider discuss options and choices with you before health care decisions are made?** |  |  |  |  |  |
| 1. **My health care provider is committed to providing the best care possible.** |  |  |  |  |  |
| 1. **My health care provider is sincerely interested in me as a person.** |  |  |  |  |  |
| 1. **My health care provider is an excellent listener.** |  |  |  |  |  |
| 1. **My health care provider accepts me for who I am.** |  |  |  |  |  |
| 1. **My health care provider tells me the complete truth about my health-related problems.** |  |  |  |  |  |
| 1. **My health care provider treats me as an individual.** |  |  |  |  |  |
| 1. **My health care provider makes me feel that I am worthy of his/her time and effort.** |  |  |  |  |  |
| 1. **My health care provider takes the time to listen to me during each appointment.** |  |  |  |  |  |
| 1. **I feel comfortable talking to my health care provider about my personal issues.** |  |  |  |  |  |
| 1. **I feel better after seeing my health care provider.** |  |  |  |  |  |
| 1. **How often do you think about changing to a new health care provider?** |  |  |  |  |  |
| 1. **How often does your health care provider consider your need for privacy?** |  |  |  |  |  |

1. **A) Are you surprised by your POC test result today?**
2. Yes
3. No
4. Prefer not to answer

**B)** Explain

1. **Would you engage in POC testing again in the future?**
2. Yes
3. No
4. Unsure

Section 4: Knowledge

|  | **True** | **False** | **I don’t know** |
| --- | --- | --- | --- |
| 1. **HCV primarily impacts the liver** |  |  |  |
| 1. **There is a vaccine to prevent HCV** |  |  |  |
| 1. **HCV can be passed through sweat, saliva or urine** |  |  |  |
| 1. **The only way to diagnose HCV is through a blood test** |  |  |  |
| 1. **People can have HCV for many years without feeling sick or experiencing symptoms** |  |  |  |
| 1. **Left untreated, HCV can result in liver failure** |  |  |  |
| 1. **There is a curative treatment available for HCV** |  |  |  |
| 1. **Once HCV has been cleared from the body, one cannot be reinfected** |  |  |  |
